# Multiomics uncovers developing immunological lineages in human 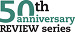


**DOI:** 10.1002/eji.202048769

**Published:** 2021-03-10

**Authors:** Emily Stephenson, Simone Webb, Muzlifah Haniffa

**Affiliations:** ^1^ Biosciences Institute Newcastle University Newcastle Upon Tyne NE2 4HH UK; ^2^ Department of Dermatology and NIHR Newcastle Biomedical Research Centre Newcastle Hospitals NHS Foundation Trust Newcastle Upon Tyne UK

**Keywords:** bioinformatics, developmental immunology, prenatal immunity

## Abstract

The development of the human immune system during embryonic and fetal life has historically been difficult to research due to limited access to human tissue. Experimental animal models have been widely used to study development but cellular and molecular programmes may not be conserved across species. The advent of multiomic single‐cell technologies and an increase in human developmental tissue biobank resources have facilitated single‐cell multiomic studies focused on human immune development. A critical question in the near future is "How do we best reconcile scientific findings across multiple omic modalities, developmental time, and organismic space?" In this review, we discuss the application of single‐cell multiomic technologies to unravel the major cellular lineages in the prenatal human immune system. We also identify key areas where the combined power of multiomics technologies can be leveraged to address specific immunological gaps in our current knowledge and explore new research horizons in human development.

## Graphical abstract

Recent advances in developmental immunology have been driven by improvements in single cell multi omic technologies, integrative bioinformatic analysis tools, and global consortia efforts. Herein, we detail these advances, which pave way for a deeper understanding of prenatal immunity and the genetic perturbations that drive disease during prenatal development. Illustration created with BioRender.com.

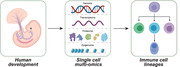

## Introduction

### A brief history of single‐cell multiomics

Single‐cell technologies are often regarded as recently developed approaches for studying cellular biology, however, we have had the ability to evaluate individual cells for centuries. During the 1800s, and with the help of the invention of the microscope, scientists described the expansion of leukocytes in disease [1]. Nowadays, many cellular characteristics at the single‐cell level can be analyzed through the use of molecular barcodes, DNA probes, and genomic technology advancements.

Multiomics refers to cellular characterization through multiple omics levels including genome (DNA), transcriptome (RNA), proteome (protein), and epigenome (open chromatin and chemical DNA modifications). Genomics was the first omics discipline to be deployed at scale to interrogate genomic polymorphisms [2], protein coding/exome sequencing [3], and next generation sequencing [4], allowing for better understanding of genetic variants associated with different medical conditions and patient prognosis.

### Single‐cell transcriptomics, epigenomics, and proteomics in the single‐cell era

Transcriptomics, the genome‐wide analysis of RNA (including transcript presence, splicing and RNA editing sites), has revealed that a large proportion of the genome is noncoding [5], and that some proportions of these noncoding regions (long noncoding RNAs) have vital roles in cellular differentiation and development [5, 6, 7]. A new frontier in omics studies is single‐cell analysis. Building upon bulk RNA‐seq, single‐cell RNA sequencing (scRNA‐seq) technologies now allow for unbiased and high‐throughput sequencing of single cells, and assignment of the barcoded mRNA molecules to specific cells within a sample [8, 9, 10]. A 2017 study redefined the repertoire of DCs and monocytes in human blood, and set the trend of atlas‐style scRNA‐seq data exploration while revealing new cell states and biology [11]. A new rare DC subset, similar to plasmacytoid DCs (pDC), was uncovered but was found to more potently activate T cells than pDCs; thus, revising the original role of pDCs. The subgroup CD1C^+^ DCs was shown to consist of two further populations and two additional monocyte fractions were also detected. Additionally, the presence of a proliferating DC progenitor within peripheral blood was identified [11]. It must be mentioned, however, that despite these exciting approaches, surveying RNA of individual cells alone is insufficient to characterize functional alterations due to post‐translational modifications.

Studying the epigenome of single cells can inform how modifications to DNA or DNA‐associated proteins facilitate cellular interactions and lineage specification [12]. Single‐cell analysis is even more crucial in this instance because the epigenomic landscape can vary considerably between cells [12]. Technologies, such as single‐cell assay, for transposable‐accessible chromatin with sequencing (scATAC‐seq) provide high‐quality accessible chromatin profiling [13] and this method coupled with combinatorial cellular indexing (sci‐ATAC‐seq) can be used for higher throughput experiments [14]. Single‐cell reduced representation bisulfite sequencing (scRRBS) is a highly sensitive epigenomic method [15] with the potential to detect the methylation status of up to 1.5 million CpG sites within the genome of an individual cell and has been used to demonstrate the importance of DNA methylation during mammalian development [15].

The field of single‐cell proteomics has historically lagged slightly behind the genomics field, due to a limit on the quantity of proteins that can be measured at any one time at sufficient sensitivity. Many of these proteomic methods rely on tagged antibodies which have been effectively used in recent studies. For example, flow cytometry can be combined with single‐cell mRNA data for protein‐level validation. Furthermore, cytometry by time of flight (CyTOF) uses heavy metal ions conjugated to antibodies, and has been used to measure the proteins of individual cord blood cells to reveal cell‐fate decisions during erythropoiesis and track changes in transcription factor expression during lineage commitment [16]. An emerging method named Single‐Cell ProtEomics by Mass Spectrometry (SCoPE‐MS) [17], utilizes tandem mass spectrometry, a technique already well‐established for bulk cell assays. The SCoPE‐MS approach is able to detect and quantify the most abundant proteins present within a single cell and has the potential to measure cells at high throughput and at a reasonable cost, thus, allowing for meaningful biological inferences [18].

### A multiomic approach to characterizing single cells

**Figure 1:**
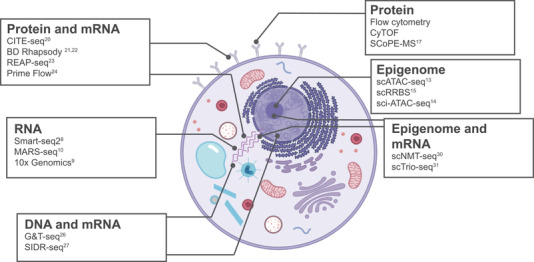
An illustration of a generic individual mammalian cell highlighting which molecules can be measured using single‐cell omic and multiomic methodologies. Examples of the methodologies are shown linked to the molecules they are able to measure. Illustration created with BioRender.com.

Studies using only one omic level of information may lack nuance, for example, a purely transcriptomic study may miss the importance of RNA/DNA/protein regulation to cell function. Multiomic technologies can be combined to fully understand cellular function (Fig. 1). They can also be used to investigate biology on a systems level by detecting interactions between cells and molecules as well as establishing regulatory networks, as reviewed in detail by Koeken et al. in this issue [19]. Combined proteome and transcriptome analyses are now common in studies aiming to provide more detailed understanding of a cell state and functional programme.

Newer techniques, such as Cellular Indexing of Transcriptomes and Epitopes by Sequencing (CITE‐seq) [20], BD Rhapsody [21, 22], RNA expression and protein sequencing assay (REAP‐seq) [23], and PrimeFlow [24] are now able to acquire both transcript and protein data from a single cell. CITE‐seq, for example, provides a digital readout of cell surface proteins by using antibody‐oligo conjugates [20]. Theoretically, using CITE‐seq, an infinite number of markers can be profiled together and allow for detection of inconsistencies between transcriptome and protein measurements. A better understanding of discrepancies between transcriptomic and proteomic profiles is an important endeavor, as inconsistencies have been reported [25] and reconciling these will clarify how far we can take transcriptome as a proxy for cellular function.

Although a combination of transcriptome and proteome approaches has been most popular in efforts toward multiomic experimental design, combinations of different omic technologies are also valuable. Combined genome and transcriptome analysis (such as using G&T‐seq [26] or SIDR [27]) allow for investigation into single‐nucleotide polymorphisms [28] and lineage tracing [29]. A technique to combine single‐cell nucleosome, methylation, and transcription sequencing (scNMT‐seq) [30] has been proposed, which could prove crucial in understanding cell ontogeny through alignment of epigenetic and transcriptomic data. Another technique, Sc‐Trio‐seq [31], that simultaneously samples the genomic copy‐number variations, DNA methylome, and transcriptome of single cells, has been used to reveal new cellular populations and examine the contribution of the genome and epigenome to the heterogeneity within populations [31].

Parallel advances in computational techniques have accompanied multiomic wet‐lab advances. For example, Marioni and team mapped gastrulation in mouse embryos at the level of RNA expression, DNA methylation, and chromatin accessibility, and further, created a statistical framework for integrating single‐cell multimodal data named MultiOmics Factor Analysis (MOFA) [32, 33]. Simultaneously, the Satija lab has updated their single‐cell analytical package, Seurat, with a newly constructed analytical framework for RNA‐protein integration using weighted‐nearest neighbor (WNN) [34]. Weighted‐nearest neighbor (WNN) has been shown to vastly improve capabilities in cell state definition leveraging RNA and protein information, particularly within lymphocytes.

## Multiomic approaches to dissect the prenatal human immune system

Human developmental research has far‐reaching impacts including innovations in tissue engineering and understanding postnatal disease. The re‐emergence of developmental signaling pathways in adult life has been shown to be a result of genetic mutations or epigenetic reconstruction that may lead to cancer [35]. The biology of stem cells present in fetal life may also shed a light on regulatory mechanisms important to regenerative medicine. Understanding the cellular and molecular programs of the developing human immune system using single‐cell multiomic technologies will generate biological insights for clinical application.

### Waves of early hematopoiesis in prenatal development

**Figure 2:**
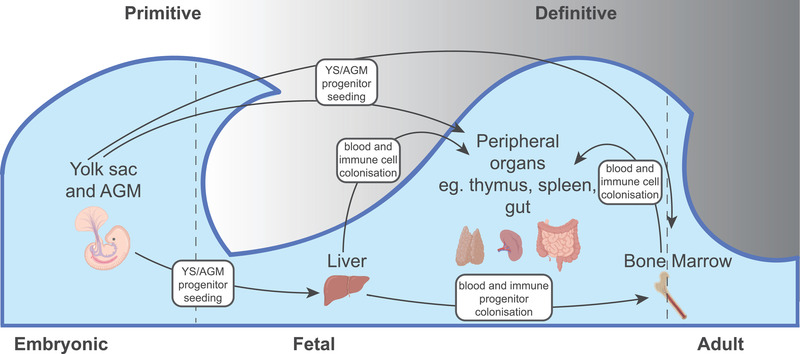
An illustration depicting the two waves of hematopoiesis during prenatal human development; the primitive wave of haematopoiesis occurs in embryonic life and the second wave of definitive haematopoiesis occurs in fetal life. During the primitive wave, progenitors from the yolk sac (YS) and aorta‐gonad‐mesonephros (AGM) seed the liver, bone marrow (BM), and peripheral tissues. During the definitive wave, blood and immune cells and progenitors further colonize firstly the BM, from the liver and then the peripheral organs from both the liver and BM. Arrows represent the movement of cells. Illustration created with BioRender.com.

The primary development of the blood and immune systems in vertebrates occurs in two waves; the primitive wave and the definitive wave [36] (Fig. 2). The primitive wave begins in the blood islands of the yolk sac (YS), with a primary function in the generation of red blood cells enabling tissue oxygenation in the developing embryo [36, 37]. In mice, this wave begins at embryonic day 7, however, in humans it begins at 2 postconception weeks (PCW) and occurs for a relatively short period of time [38]. During the primitive wave, YS progenitors are thought to give rise to endothelial cells and early hematopoietic progenitors [39].

At 3‐4 PCW, the first intraembryonic progenitors are formed in the aorta‐gonad‐mesonephros (AGM); a region of the embryonic mesoderm that transiently gives rise to blood and immune cells [38]. YS and AGM‐derived progenitors subsequently seed tissues with roles important to hematopoiesis, namely, the fetal liver (FL) and fetal BM [38]. The switch from primitive to definitive hematopoiesis coincides with a switch in the major site of blood and immune cell development from the YS to the FL; the FL is the major hematopoietic organ in the second trimester and is known to have an important role in erythropoiesis [38, 40].

It is not yet established how hematopoietic stem cell (HSC) expansion and differentiation is supported in the FL but interestingly, the HSC pool expands rapidly in the FL, compared to the BM where HSCs are mostly quiescent [41]. Mouse studies have informed us that over gestational time, the main focus of hematopoietic differentiation changes. Early on, the FL is rich in CFU erythroids and proerythroblasts, suggesting active definitive erythropoiesis, however, myeloid and lymphoid progenitors seem to accumulate with developmental age [42].

The fetal BM is then colonized by blood and immune progenitors at around 11 PCW and becomes the dominant site of hematopoiesis after 20 PCW and into adulthood [38]. All blood and immune cells derived from the AGM, YS, FL, and fetal BM go on to seed peripheral organs, such the spleen (the site of B‐cell maturation), the thymus (site of T‐cell selection), and nonlymphoid tissue, including the gut, where they undergo maturation programmes [43, 44] (Fig. 2). The further rise of these specific immunological lineages is supported by their site of development [45].

### Hematopoietic stem cells and progenitors

A study by Notta et al. profiled 3000 stem and progenitor cells using a variety of single‐cell methods including scRNA‐seq, single‐ cell in‐vitro culture, and flow cytometry [46]. They profiled cells from the FL, cord blood, and adult BM. Groups of cells that were previously thought to be homogenous were further purified and their differentiation potency was analyzed. Both stem and progenitor cells in the FL had oligopotent potential compared to adult BM which showed multipotency restricted only to HSCs. Our group expanded on this observation in the FL, demonstrating that FL HSC differentiation potential rapidly changed across gestational stages in utero [40]. Through single‐cell transcriptome data, we revealed three intermediate cell states between FL HSCs, multipotent progenitors, and differentiated mononuclear phagocytes. The intermediate states include a neutrophil–myeloid progenitor, a monocyte precursor, and a DC precursor [40].

It is also important to note the value of reanalyzing the increasing numbers of transcriptome datasets (which also has cost‐related benefits) for further biological insights. Granulocyte‐monocyte precursors (GMPs) from published murine single‐cell transcriptomic datasets [47, 48] have been reinvestigated in this way, with Kwok et al. proposing that GMPs contain a mixed population of monocyte, neutrophil, eosinophil, and basophil progenitor subsets [49], supporting the theory that GMPs were a combination of unipotent progenitors [47], in contrast to earlier theories which proposed oligopotent monocytic and neutrophil potential of GMPs [50]. Underscoring the importance of orthogonal technologies, this study relied upon flow cytometry to validate the heterogeneity of GMPs and to provide putative markers for the neutrophil progenitor, which was invaluable as transcriptome data cannot reliably provide cell‐surface markers.

### Mononuclear phagocytes

The mononuclear phagocyte system (MPS) is composed of monocytes, macrophages, and DCs that link the innate and adaptive immune systems. Murine fate mapping and in‐vitro studies have been the basis of our knowledge of the lineages of the MPS. These studies have also revealed important differences of these lineages in early development and the adult equivalent [51].

#### Monocytes

Our understanding of human fetal monocyte functionality is currently limited. Using single‐cell transcriptomics, we have recently shown that they do correlate with classical adult monocytes [40]. Additionally, we only observe one fetal kidney monocyte population compared to two populations in the adult kidney [52]. In the mouse, monocytes differentiate in the FL from erythromyeloid precursors originating in the YS [53]. This is not yet established in human development and requires further investigation.

#### Dendritic cells

In adult life, DCs are involved in the initiation and regulation of adaptive immune responses and are essential for establishing immunological memory. However, during prenatal development DCs have a role in building tolerance [54]. The DC lineage is generally reported to be HSC dependent [55], with conventional DCs (DC1 and DC2) and pDCs detected in the fetal spleen, skin, lung, and thymus [54, 56]. Further to this, we have now detected DCs (DC1 and DC2) in the FL from as early as 6 PCW, prior to the establishment of the BM [40]; this is not necessarily surprising considering murine DCs can be generated in vitro from FL progenitors [57]. Whether the FL‐derived DCs are the same as those generated from BM progenitors is unknown. A cross‐tissue multimodal analysis, such as CITE‐seq and ATAC‐seq, would enable us to better characterize the cellular phenotypes and differentiation requirements, including gene regulatory networks, in more detail than transcriptome measurements alone.

#### Macrophages

Macrophages in adults can originate either from prenatal YS‐derived progenitors or BM HSC/progenitors via a monocyte intermediate state. This is reflected in their diverse range of functions including regulating homeostasis, chaperoning the maturation of fetal tissues, and their well‐known roles in immunity [58]. As mentioned previously, murine studies have been invaluable in improving our understanding of macrophage ancestry and recent investigations have displayed evidence that they are one of the very first immune cells to emerge in the human YS at around Carnegie stage 11 [59].

Bian et al. used scRNA‐seq to study the ontogeny of YS‐derived macrophages in detail and identified two HSC‐independent pathways of macrophage development [59]. They profiled CD45 positive cells from different anatomical locations including the embryonic YS and fetal blood, skin, liver, lung, and head. Trajectory analysis revealed the dual origin of tissue‐resident macrophages; an early population found within the YS and a population found later in gestation developing through a monocyte intermediary [59]. These observations follow what we have already established in mouse and zebrafish development [60, 61].

Additionally, fetal macrophages express specific tissue markers, such as *VCAM1* in liver and *F13A1* in skin, compared to other cells of the MPS [40], another characteristic which is also identified in mice [62].

### Innate lymphocytes

Innate lymphoid cells (ILCs), including NK cells, are heterogeneous and plastic in steady state as well as in disease. Their characterization has been aided by single‐cell sequencing studies [63]. During fetal life, as well as ILCs, lymphoid tissue inducer (LTi) cells are present and essential for the development of secondary lymphoid tissue [64]. The definitive lineage relationship between ILCs and LTi cells is poorly understood in humans. Single‐cell multiomic studies in mice have demonstrated the bifurcation of these lineages which is controlled by the induction of transcription factors *PLZF* and *TCF1* [65]. Analyses using flow cytometry and single‐cell culture established this divergence from lymphoid progenitors isolated from the mouse FL [65]. Single‐cell transcriptomics done in parallel provided a detailed map of the transcriptional regulation of this process, however, further investigations are needed to validate these findings [65].

Additionally, Baerenwaldt et al. illustrated the role of Flt3L and IL‐7 in the development of ILCs in mice. Flt3L appeared to be essential for early differentiation of ILCs from progenitors cultured from the FL, whereas IL‐7 was more important for the development of ILCs in peripheral organs such as the spleen [66]. It would be compelling to investigate this in humans using single‐cell technologies.

### Adaptive lymphocytes

#### Early studies into T‐ and B‐lymphocyte function

The distinction between antibody‐producing lymphocytes and thymus‐derived lymphocytes was first proposed in 1965, through convergent experimentation in birds and mammals [67, 68]. Since a landmark 1974 study in murine FL and fetal BM, BM‐derived B‐ lymphocytes are known to have origin in prenatal life [69, 70, 71]. Early thymectomy studies in mice also hint to prenatal origins of T lymphocytes as early as 1962 [72]. These studies paved the way for the investigation of function of B and T cells during human fetal development.

#### B‐lymphocyte differentiation and selection in fetal life

The molecular regulation of B‐lineage commitment (also termed B‐lymphopoiesis) has been investigated in adult mice at the single‐cell resolution using FACs, microarray, and single‐cell culturing [73, 74], and in human adult tonsil using transcriptomic and BCR‐enriched scRNA‐seq [75]. pro B progenitors have been shown to drive B‐lineage differentiation in the human FL as early as 7 PCW, with further waves of B lymphopoiesis initiated in the human fetal BM weeks later [40, 76, 77]. The BM becomes a dominant source of mature naive B cells during mid‐gestation, which are then thought to seed the spleen for selection [76].

#### T‐lymphocyte differentiation and selection in fetal life

Double positive, proliferating, mature, and recombining thymocytes cells have been identified in adult mice using single‐cell transcriptomics [78], in a study which showed, for the first time, that MHC class II is not restricted to professional APCs in mice (and is expressed in thymocytes). Treg and memory cells have also been investigated to a high resolution in mice using single‐cell transcriptomics [79].

In the context of human life, T‐cell heterogeneity in lungs, lymph nodes, BM, and blood have been profiled in both health and disease using scRNA‐seq [73]. In the context of human fetal development, cellular lineages contributing to and constituting human thymus‐derived lymphocytes have been comprehensively characterized at single‐cell resolution in the AGM [40, 80], FL [80] and fetal and pediatric thymus [80, 81]. These studies reveal that the presence of early thymic progenitors (ETP) in the AGM and FL, the egress of early thymic progenitors (ETP) from FL to thymus, more than 50 cell states present in human thymus, dynamic changes in abundance and transcriptomic profile of thymic populations across gestation, novel stromal populations in thymus, and strong bias in human VDJ usage [81].

#### Focus of future multiomic studies on adaptive lymphocytes

It would now be timely to generate multiomic maps of organs implicated in B‐cell and T‐cell differentiation (such as the fetal BM and fetal spleen), in order to further understand the adaptive immune response and its formation during healthy human development. We could answer specific questions such as which B or T cell states are present throughout fetal organs over gestation and what, if any, functionality they have. It would also be advantageous to know from a clinical perspective how lymphoid receptor (BCR/TCR) clonal expansion is patterned across fetal organs during human development, and if detected, what utility clonality offers prior to antigen challenge.

Recognizing the genetic basis of diseases implicated in dysregulation of B or T lymphopoiesis could have an impact on clinical therapy. For example, in B‐cell acute lymphoblastic leukemia, which is thought to have origins during early development. Transcriptomic studies could be built upon to further develop in‐vitro organoid culture models of human in‐vivo thymic tissue. Transcriptomic data could also be reanalyzed with additional chromatin and/or proteome data to help further understand the role of thymus in acquired and congenital T‐cell deficiency syndromes such as Wiskott–Aldrich syndrome.

## The future of multiomic technologies

### Current challenges in the field of single‐cell multiomics

Approaches for multiomic data integration (including wet lab and computational) are constantly improving, and it is now becoming conceivable that, with added scalability, we could create cellular atlases of organs with a multiomic level of detail. Beyond this point, fundamental questions will remain, for example: (1) How well do multiomic profiles of isolated cells match up to those in an in‐vivo environment? (2) How does stochasticity of gene expression and protein influx/efflux affect (or otherwise bias) our understanding of cellular function? (3) How can we best create a data visualization platform that is both intuitive to scientists and nonscientists, and able to represent data at multiple omics levels simultaneously (including, perhaps, the additional dimension of spatiality)?

### Complementary approaches to understand human development

Distilling the intricacies of immunopoiesis during human development cannot be done with multiomic approaches alone; a concert of complementary techniques must also be incorporated into experimental plans. By design, omic studies rely upon sampling of accessible material, a resource which is not available at the earliest stages of human development such as the preimplantation stage. This limitation can be overcome through use of in‐vitro models, which, alongside organoid and animal models, have the added benefit of being amenable to genetic and molecular perturbation. These complementary techniques facilitate mechanistic studies and lineage mapping, and allow for development to be studied in real time as opposed to the "snapshot" time‐series nature that many multiomic experiments have.

### Leveraging international consortium efforts will be vital in overcoming challenges

Human developmental research has benefited from a recent surge in resolution, facilitated by previously described technological advancements in single‐cell omics, combined with improved access to human embryonic and fetal material. Access to research material has greatly improved thanks to tissue repositories supporting human development research, such as the Human Developmental Biology Resource (HDBR: www.hdbr.org), the largest fetal tissue bank in the UK.

International ventures, such as HDBR and its equivalents, allow researchers to expand beyond animal models and in‐vitro culture models. Although these are valuable particularly for early developmental stages, when samples are inaccessible and are amenable to perturbation and genetic manipulation, there are species‐specific differences which must be resolved [82]. The integration of multiomic techniques to map whole human embryos and fetuses is currently being pioneered in the context of human development by the Human Development Cell Atlas (HDCA) initiative, part of the Human Cell Atlas project [83].

The HDCA aims to "generate a comprehensive profile of all cell types and states present during development" using molecular and spatial methods to provide a 3D atlas. The HDCA has already published atlas type works on organs implicated in immunopoiesis, such as YS and FL [40] and fetal and pediatric thymus [81]. Research efforts on nonlymphoid tissues also contribute to broader understanding of the migration and function of immune cells during development (for example, a recent scRNA‐seq study on the developing limb [84]). The completion of an HDCA will help us to understand how immunity is established and maintained in health, and may help us to uncover previously unknown roles for blood and immune cells.

### Concluding remarks

Multiomics has shown great potential in the analyses of developing human tissues. We are now in an era where we can survey multiple molecules from individual cells on an enormous scale, providing a level of detail previously unimaginable. These new omics studies have identified new cell types within lineages and revealed new roles for known cells. Large scale and high throughput profiling of cells, tissues, and organ systems will discover emergent properties that we would not have exposed by looking at individual lineages. To uncover the ontogeny of the immune system in great detail will allow us to demonstrate how genetic perturbations drive disease, especially those with origins during prenatal development. Global consortia efforts will be crucial to successfully deliver such analyses at this scale.

## Conflict of Interest

The authors declare no financial or commercial conflict of interest.

**Abbreviations**
AGM: aorta‐gonad mesonephros
CITE‐seq: cellular indexing of transcriptomes and epitopes by sequencing
FL: fetal liver
GMP: granulocyte‐monocyte precursors
HDBR: human developmental biology resource
HDCA: human development cell atlas
HSC: hematopoietic stem cells
ILCs: innate lymphoid cells
LTi: lymphoid tissue inducer
MPS: mononuclear phagocyte system
PCW: postconception weeks
pDC: plasmacytoid DCs
REAP‐seq: RNA expression and protein sequencing assay
scATAC‐seq: single‐cell assay for transposable‐accessible chromatin w/sequencing
sci‐ATAC‐seq: single‐cell combinatorial indexing ATAC‐seq
scNMT‐seq: single‐cell nucleosome, methylation and transcription sequencing
SCoPE‐MS: single‐cell proteomics by mass spectrometry
scRRBS: single‐cell reduced representation bisulfite sequencing
YS: yolk sac

## Data Availability

Data sharing is not applicable to this article as no new data were created or analysed in this study.
